# Analysis of the activation routes induced by different metal oxide nanoparticles on human lung epithelial cells

**DOI:** 10.4155/fso.16.2

**Published:** 2016-04-15

**Authors:** Rosana Simón-Vázquez, Tamara Lozano-Fernández, Angela Dávila-Grana, África González-Fernández

**Affiliations:** 1 Immunology, Biomedical Research Center (CINBIO) & Institute of Biomedical Research of Orense, Pontevedra and Vigo (IBI), University of Vigo, Campus Lagoas Marcosende, Vigo, Pontevedra, 36310 Spain

**Keywords:** cytokines, inflammation, MAPK, metal oxide nanoparticles, NFκB

## Abstract

Nanoparticles (Nps) can induce toxicity in the lung by accidental or intentional exposure. The main objective of the study reported here was to characterize the effect that four metal oxide Nps (CeO_2_, TiO_2_, Al_2_O_3_ and ZnO) had at the cellular level on a human lung epithelial cell line. This goal was achieved by studying the capacity of the Nps to activate the main mitogen-activated protein kinases (MAPKs) and the nuclear factor NFκB. Only ZnO Nps were able to activate all of the MAPKs and the release of Zn^2+^ ions was the main cause of activation. ZnO and Al_2_O_3_ Nps activated the NFκB pathway and induced the release of inflammatory cytokines. CeO_2_ and TiO_2_ Nps were found to have safer profiles.

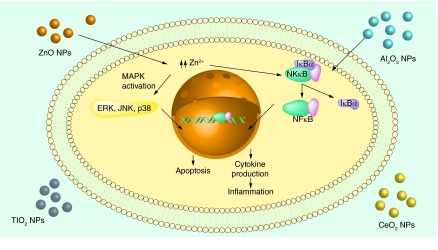

The graphical abstract was obtained using Servier Medical Art.

## Abstract (lay abstract)

**Lay abstract:** When cells are exposed to a stimulus, they can activate different signaling pathways and these lead to different responses such as proliferation, differentiation, migration or inflammation. The objective of the work described here was to characterize the effects of several metal oxide nanoparticles at the cellular level by studying their capacity to activate the main mitogen-activated protein kinases (MAPKs) and the expression of the transcription factor NFκB on a human lung epithelial cell line. These signaling proteins play a relevant role in the vast majority of the cellular events that are triggered in eukaryotic cells after any stimulus.

**Figure 1.**
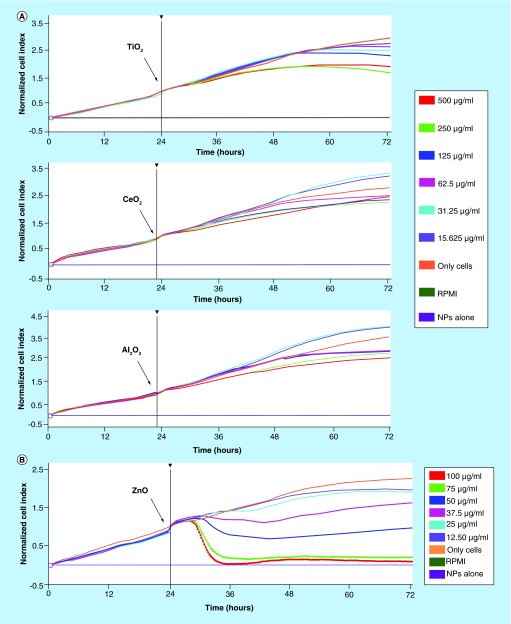
**Effect of Nps on the viability of NCI H460 cells.** Cells were allowed to grow until they reached the exponential phase. **(A)** TiO_2_, CeO_2_ and Al_2_O_3_ NPs were added (indicated with an arrow) at different concentrations: 15.6 (violet line), 31.25 (blue line), 62.5 (pink line), 125 (dark blue line), 250 (green line) and 500 μg/ml (red line). **(B)** ZnO Nps were added (indicated with an arrow) at different concentrations: 12.5 (violet line), 25 (blue line), 37.5 (pink line), 50 (dark blue line), 75 (green line) and 100 μg/ml (red line). The calculated LD50 was around 50 μg/ml.

**Figure 2.**
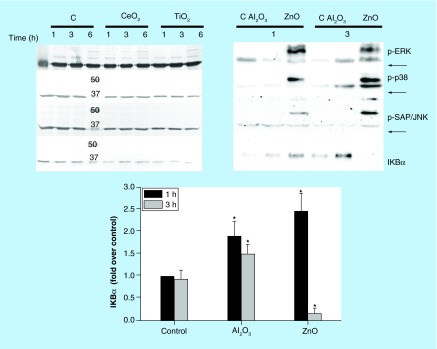
**Activation of the MAPK (p-ERK1/2, p38 and p-SAPK/JNK) and the NFκB pathways induced by CeO_2_, TiO_2_, Al_2_O_3_ and ZnO Nps in the NCI-H460 lung cell line.** The activation of the MAPK and NFκB pathways was studied by western blot. The expression of phosphorylated (p) proteins (p-ERK1/2, p-38 and p-SAP/JNK) is indicated at different time points (1, 3 and 6 h). All Nps were tested at 100 μg/ml, except for ZnO (50 μg/ml). The numbers in the figure correspond to the molecular weight of the protein marker and GAPDH was used as a loading control (bands indicated with arrows). The activation of the NFκB pathway was analyzed as the degradation of the IκBα inhibitor by western blot and normalized to the controls (C, untreated sample) at different times (1, 3 h). *Statistically significant differences (p < 0.05) in the protein level compared with the control.

**Figure 3.**
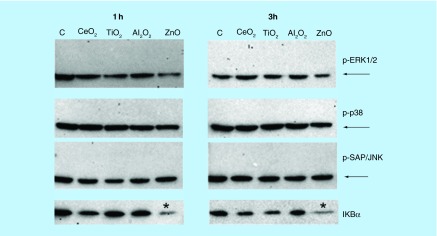
**Expression of the MAPK and IκBα protein in NCI-H460 cells incubated with low concentrations of CeO_2_, TiO_2_, Al_2_O_3_ and ZnO Nps.** The activation of the MAPK and NFκB pathways was studied by western blot at two different time points (1 and 3 h). All Nps were tested at 10 μg/ml, except for ZnO Nps (5 μg/ml). The expected bands corresponding to the phosphorylated p-ERK1/2, p-38 and p-SAP/JNK are indicated, but they were not detected in the NCI-H460 cell line at these Np concentrations. GAPDH was used as a loading control and the bands are indicated with arrows. The activation of the NFκB pathway was analyzed by the degradation of the IκBα inhibitor. *Statistically significant differences (p < 0.05) in the protein level compared with the control.

**Figure 4.**
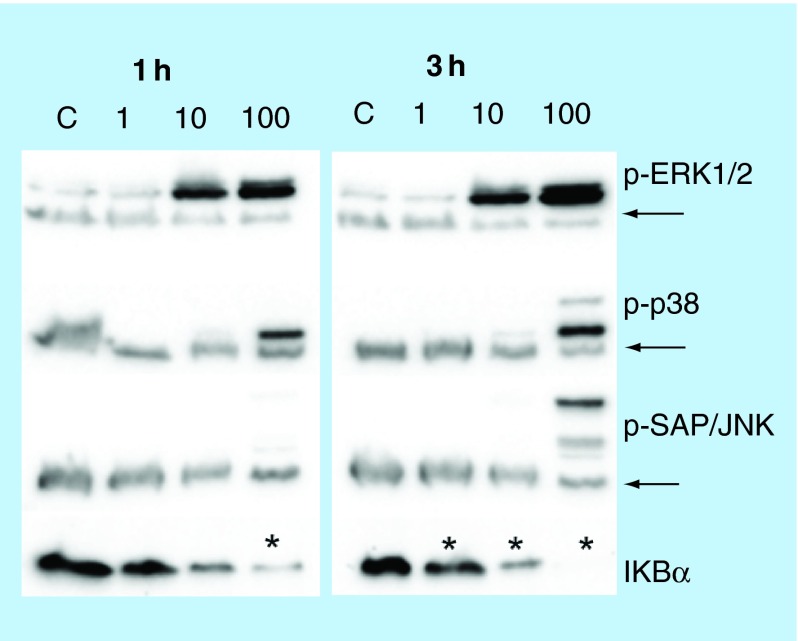
**Influence of the Zn^2+^ ion concentration on the activation of the MAPK and NFκB pathways in NCI-H460 cells.** The activation of the MAPK and NFκB pathways in NCI-H460 cells was studied by western blot at two different time points (1 and 3 h) and at three different concentrations (1, 10 and 100 µg/ml) of Zn^2+^ ions. The expression of phosphorylated (p) proteins (p-ERK1/2, p-38 and p-SAP/JNK) is indicated along with the degradation of the IκBα inhibitor. GAPDH was used as a loading control and the bands are indicated with arrows. Statistically significant differences (p < 0.05) in the protein level compared with the control.

## Background

Nanoparticles (Nps) can reach the lung by intentional administration or accidental exposure, either by inhalation or systemic administration [1]. Accidental inhalation could occur through exposure to nanoparticle aerosols formed during the manufacture, manipulation or packaging of these nanomaterials [2]. The tendency of the Nps to form bigger agglomerates, which can reach the micron size, decreases the risk of inhalation. However, surface modification of the Nps to avoid agglomeration, such as the use of polyethylene glycol or protein binding, decreases the tendency to agglomerate [3]. In addition, the inhaled Nps could reach not only the lung but other organs, including neurons by translocation through the olfactory bulb, or the blood through interstitial translocation, followed by their systemic distribution [3]. In contrast, systemic delivery by injection of the Nps or by absorption following dermal application or ingestion may cause incidental pulmonary exposure [1].

Several toxicological studies on inhaled metal oxide Nps in animals have been carried out and numerous differences between the different animal models were identified for the same type of Nps. For instance, the inhalation of ultrafine TiO_2_ particles produces different effects in mice, rats and hamsters, with rats affected the most by inflammation, followed by mice and hamsters [4]. The latter group showed a very fast particle clearance compared with the other two species’. In contrast, the results of another study showed that fine and ultrafine TiO_2_ Nps appear to be safe for mammals and aquatic organisms following acute exposure [5]. Specifically, *in vivo* pulmonary toxicity studies in rats carried out with the aforementioned Nps demonstrated the low inflammatory potential and low lung tissue toxicity. The results of a different study showed that the subacute inhalation of TiO_2_ Nps caused moderate inflammation in mice but this was resolved within 3 weeks [6]. Nevertheless, the murine model is not the most appropriate to describe lung toxicity because significant differences with primates are found in the mechanism of toxicity and, hence, in the outcomes of the exposure [7].

The results of human toxicological studies have shown that sustained exposure to Nps can cause severe inflammation, with pleural effusion, pulmonary fibrosis, granuloma and impairment of the breathing function, as observed in a group of young female Chinese workers accidentally exposed to polyacrylate Nps over several months. As a result of this strong lung dysfunction, two of the workers died shortly after the onset of the disease [8].

Nps entering via the respiratory tract could be responsible for numerous toxicological events. The main underlying cellular mechanisms of Np-induced toxicity are the ineffective clearance of the Nps, oxidative stress and genotoxicity [9]. The increase in levels of the reactive oxygen species’ (ROS) could lead to the activation of several signaling pathways, such as the MAPK and the expression of inflammatory cytokines [10–12]. Genes involved in lung inflammation are transcribed as a result of this activation. Np-induced genotoxicity could be responsible for DNA damage in cells and tissues, altered cell cycle kinetics, induced expression of p53 and DNA repair related proteins, mutagenesis and carcinogenesis processes [13]. Other lung disorders, in addition to inflammation, could be induced by exposure to the Nps and these include fibrosis, pneumoconiosis and exacerbation of asthma [9]. Moreover, an association between the inhalation of particulate matter and an increase in pulmonary and cardiovascular morbidity and mortality has been established [14].

In general, metal oxide Nps have been shown to induce low inflammatory cytokine release *in vitro* in airway cells (BEAS-2B) compared with particles derived from soil dusts, and they are probably less toxic to the lung [15].

In addition to the well-characterized cytotoxicity, ROS production and genotoxicity, metal oxide Nps may induce other effects on the cells after interaction and/or internalization. As a consequence, characterization of the MAPKs and the NFκB pathways could provide more detailed information and allow discrimination between those Nps that induce some cellular effect and those that are more innocuous.

MAPK and NFκB are well-known signaling proteins that are activated by several extracellular stimuli and they induce a broad spectrum of cellular effects, such as proliferation, differentiation, migration, inflammation and apoptosis, among others.

The specific activation of the three main MAPKs (ERK, p38 and SAP/JNK) and their relation with pathogenic effects are of great interest. For instance, the activation of ERK is mainly related to proliferation while the activation of SAP/JNK is related to apoptosis, as observed with ultrafine carbon particles in rat lung epithelial cells depending on the dose and time [16]. The activation of MAPK is also relevant in the carcinogenesis process in asbestos-induced toxicity in smokers. Both toxins, in other words, cigarette smoke and asbestos, induce the activation of MAPK and the expression of AP-1 transcription factor regulated genes [17].

MAPK signaling can be triggered by activation of tyrosine kinase membrane receptors, such as the EGFR, by ligand binding or by oxidative stress via several different mechanisms [18,19].

The NFκB family of transcription factors (TFs) are also key regulators of immune, inflammatory and acute phase responses, and these TFs are also implicated in the control of cell proliferation, apoptosis and oncogenesis [20]. These TFs also play a key role in the induction of pro-inflammatory gene expression, leading to the synthesis of inflammatory cytokines such as IL-6, IL1β and TNF-α, chemokines such as IL-8, adhesion molecules such as ICAM-1, growth factors and enzymes [21].

NFκB plays a major role in common lung diseases associated with a relevant inflammatory component, such as asthma and chronic obstructive pulmonary disease (COPD) [21–23]. In COPD, the activation of NFκB has been implicated in the pathogenesis, but its exact role is not clear due to the heterogeneity of the patient population. NFκB activation has also been related with mineral dust diseases and it is probably involved in their pathogenesis [21].

Several studies have already been conducted into cellular toxicity induced by metal oxide Nps in different cell types mediated by the activation of those pathways. For instance, it has been shown that ZnO Nps induce apoptosis by p53-p38 activation in a human dermal fibroblast cell line [24] and CeO_2_ Nps are able to induce the activation of the three MAPKs (ERK1/2, p38 and JNK) in a hepatocyte cell line, thus increasing the ROS levels and reducing the viability of the cells [25]. In fact, this activation and the cellular effects were attenuated by using an antioxidant. Similar cellular outcomes were found for silica Nps in HUVECs, a human umbilical vein endothelial cell line, where the activation of JNK, p53 and NFκB was also mediated by oxidative stress, which induces inflammation and apoptosis in the cells exposed to the Nps at a concentration higher than 50 µg/ml [26]. In contrast, TiO_2_-activated neutrophils in a concentration-dependent manner mediated by the activation of ERK and p38. These particles also inhibited apoptosis in a concentration-dependent manner at concentrations of 20 µg/ml or above and, after a long incubation time, they induced the production of several cytokines such as IL-8. In fact, TiO_2_ Nps exerted several neutrophil agonist functions *in vitro* [27]. Correspondingly, in a bronchial epithelial cell line TiO_2_ Nps also induced the expression of IL-8 and once again this was mediated by the activation of ERK and p38, as determined using inhibitors of these kinases that reduced the expression of this inflammatory cytokine [28]. More recently, this TiO_2_-mediated IL-8 release was also detected in human colon epithelial cells together with NFκB activation [29]. The fact that the activation of two different pathways may lead to the same cellular event could be due to crosstalk between them [30]. However, this behavior could also be due to a different triggering event or selection of a different signaling pathway depending on the stimulus, as observed in normal human bronchial epithelial cells incubated with ultrafine carbon particles. The IL-8 expression induced by these particles was mediated by p38 and not by NFκB, behavior that has been described previously for those cells [31] and has also been observed with ZnO Nps [32].

In the study described here, we compared the activation of MAPK and NFκB pathways induced by four different metal oxide Nps (CeO_2_, TiO_2_, Al_2_O_3_ and ZnO) in a non-small-cell lung epithelial cell line: NCI-H460. Two different concentrations per Np were tested: 10 and 100 µg/ml for CeO_2_, TiO_2_ and Al_2_O_3_ Nps; but only 5 and 50 µg/ml for ZnO Nps (due to their high cell toxicity [33]). Due to the association proposed between ion release and the toxicity induced by some metal oxide Nps [12,34–37], the potential effect produced by Zn^2+^ ions was also evaluated by adding ZnCl_2_ salt to the cells. Finally, the cytokine release induced by the metal oxide Nps was characterized in human peripheral blood mononuclear cells (PBMCs).

## Methods

### Nanoparticle preparation

The commercial Nps used in this work were supplied by Sergio Moya (CIC-Biomagune, San Sebastian, Spain) as part of the HINAMOX project (7th EU framework program) and they were: TiO_2_ Nps (3.59 ± 0.94 nm, anatase phase) and Al_2_O_3_ Nps (13.56 ± 8.37 nm) from PlasmaChem (Berlin, Germany); CeO_2_ Nps (13.04 ± 12.13 nm) and ZnO Nps (36.16 ± 18.27 nm) from Evonik Industries AG (Essen, Germany). The Np size was determined by TEM and all the Nps were uncoated. The TEM images and DLS characterization in water were described previously [38]. The DLS measurements in culture medium RPMI 1640 from Gibco, Life Technologies (CA, USA) supplemented with 10% fetal bovine serum (FBS) were performed using a Zetasizer Nano ZS from Malvern Instruments (Worcester, UK) at 25°C. Suspensions of Nps at 1 mg/ml in culture medium were prepared for zeta potential determination. Each sample was measured four-times, with a combination of 200 runs per measurement. For zeta size determination, Nps (25 µg/ml) were suspended in medium and three measurements were averaged, with a combination of 13 runs per measurement. The Np size distribution was determined by intensity.

A stock of Nps was prepared at 10 mg/ml in milli-Q water and the mixture was sonicated for 10 min at low frequency (47 kHz) in a Branson 1510 ultrasonic bath (CT, USA). After this time 10% fetal bovine serum (FBS: PAA Laboratories, Pasching, Austria) was added. Finally, the Nps were diluted to the working concentration in RPMI 1640 supplemented with 10% FBS. The sterility of the Nps was preserved in all cases and for control samples the same amount of water with 10% FBS was added.

### Cell viability assay: xCELLigence^®^ system

The effects of the different NPs on cell viability were studied using the xCELLigence RTCA DP from Roche (Basel, Switzerland). This system uses plates that contain a gold electrode at the bottom of each well. This electrode can measure the changes in impedance during cell growth in real time, and the method is only suitable for adherent cells.

In this case, NCI H460 cell line was cultured at 1 × 10^4^ cells per well (37°C and 5% CO_2_) in 200 µl of RPMI 10% FBS and the sample was incubated for 24 h, after which the exponential phase had been reached. The medium was removed and 200 µl of each treatment were added. The NPs were tested at six different concentrations by performing serial dilutions (1:2) starting from 500 µg/ml. In the case of ZnO Nps, the starting dilution was 100 µg/ml in order to calculate an accurate LD50. Culture medium and NPs alone at the highest concentration were used as negative control. The whole experiment lasted 72 h and the impedance was monitored at 15-min intervals. The LD50 values were calculated using RTCA Software 1.2.1.

### Cell culture & incubation with the nanoparticles

Signaling pathway activation assays were performed on the human tumoral NCI-H460 cell line, which was purchased from ATCC (American Type Culture Collection, Manassas, VA, USA). Cells were cultured at 37°C in 5% CO_2_ with RPMI 1640 medium supplemented with 10% FBS and Penicillin-Streptomycin (Gibco, Life Technologies, CA, USA). The NCI-H460 lung adenocarcinoma cell line was seeded at a density of 5 × 10^6^ in 25 cm^2^ culture flasks (Falcon™ BD Biosciences, NJ, USA). Except for ZnO (used at 5 and 50 µg/ml due to its high cytotoxicity), Nps were added to the cells at concentrations of 10 and 100 µg/ml. The influence that Zn^2+^ ions had on ZnO Np-induced cell activation was also studied with a stock of 10 mg/ml ZnCl_2_ (Sigma-Aldrich Co. LLC, MO, USA) prepared in milli-Q water. The stock solution was added to the cells to give final Zn^2+^ concentrations of 1, 10 and 100 µg/ml.

The cells were incubated with the Nps for different times and they were then washed with phosphate-buffered saline (PBS).

### Study of the MAPK & NFκB activation pathways

Cell extracts were prepared in a lysis buffer with 10 mM Tris.HCl [pH 8], 150 mM NaCl, 2.5 mM EDTA, 1% NP-40 and a protease and phosphatase inhibitor cocktail (Complete Mini and PhosphoStop from Roche Ltd., Basel, Switzerland). The cell lysates were centrifuged (16,000 × *g* at 4°C for 5 min) in an Eppendorf 5415 R centrifuge (Eppendorf AG, Hamburg, Germany) to remove the cell residue and Nps. Cell extracts were resolved on 10% SDS-PAGE gels and transferred to polyvinylidene difluoride (PVDF) membranes (Immun-BlotTM 0.2 µm, BioRad Laboratories, Inc., CA, USA). The membranes were washed with Tris buffer saline with 1% Tween-20 (TBST) and blocked with 5% skimmed milk (Sigma-Aldrich Co.) in TBST for 1 h at room temperature.

The phosphorylation of ERK1/2, p38 and SAPK/JNK kinases was determined in western blots probed with specific rabbit monoclonal anti-human p-ERK1/2, p-p38 and p-SAPK/JNK antibodies against the phosphorylated proteins diluted 1:10,000–1:20,000 in TBST. All antibodies were purchased from Cell Signaling Tech (MA, USA). Anti-GAPDH rabbit polyclonal antibodies were used at 1:80,000 as internal control (Sigma-Aldrich Co. LLC).

NFκB activation was assessed through the degradation of the IκBα inhibitor in western blots probed with an anti-IkBα monoclonal antibody diluted at 1:10,000. Due to overlap with the GAPH protein, the value used to normalize the results for the IκBα protein was the mean intensity of the GAPDH obtained in the other western blots, taking into account the fact that the same volume of protein extract was loaded for each sample.

Goat anti-rabbit HRP-conjugated IgG antibodies diluted 1:50,000 in TBST with 2.5% skimmed milk were used as secondary antibodies. Antibody binding was detected with the Clarity™ Western ECL Substrate, and the protein bands were analyzed and quantified using the ChemiDoc XRS imaging system, both from Bio-Rad Laboratories (Hercules, CA, USA). Three independent experiments were performed for each condition and a one-way ANOVA was performed to test the homogeneity of the variances, followed by a Dunnett's T3 or a Tukey's statistical test to compare the treated samples with the control (untreated sample): statistical significance (p <0.05). In the cases where statistical studies were not carried out, two or three independent experiments were performed.

### Cytokine detection in human peripheral blood mononuclear cells

The release of cytokines was studied in PBMCs from three different healthy donors treated independently. The PBMCs were obtained by density gradient using Ficoll-Paque PLUS™ from GE Healthcare (Uppsala, Sweden). Briefly, EDTA anticoagulated blood was diluted with an equal volume of PBS and added to Ficoll in a 7:3 ratio (diluted Blood:Ficoll) and centrifuged at 180 × *g* for 30 min at 20°C. The mononuclear cells, which were located at the interface between Ficoll and plasma, were collected with a Pasteur pipette and washed twice with complete medium by centrifugation at 100 × *g* for 5 min at 20°C.

Then 1 × 10^5^ hPBMCs were incubated in 96-well plates in the presence of the NPs at two different concentrations: 20 and 200 µg/ml. As a positive control, cells were incubated with a combination of lipopolysaccharide (LPS) from InvivoGen (CA, USA) and phytohemagglutinin (PHA) from Sigma-Aldrich (MO, USA) at 1 and 10 µg/ml, respectively. Culture medium was used as a negative control.

After 24 h of incubation at 37°C with 5% CO_2_, the plate was centrifuged (100 × *g*, 5 min, 4°C) and the supernatant was collected and stored at -20°C prior to analysis. The concentrations of 11 different cytokines (IFN-γ, IL-1β, IL-2, IL-4, IL-5, IL-6, IL-8, IL-10, IL-12 p70, TNF-α, TNF-β) were determined using the FlowCytomix™ Th1/Th2 kit from eBioscience (CA, USA) according to the manufacturer's instructions. This kit allows the simultaneous detection of different cytokines as it is based on antibody-conjugated beads that can be differentiated by their sizes and by their distinct spectral ranges. Finally, the samples were studied by flow cytometry (FC500, Beckman-Coulter; FL, USA) and data were analyzed with the FlowCytomix Pro 3.0 Software (eBioscience, San Diego, CA, USA).

The panel of cytokines studied was divided into type 1 cytokines, including those cytokines that induce a Th1 or cellular response, and type 2 cytokines, which are released in a Th2 or humoral response. Other cytokines were also evaluated such as IL-8, a cytokine that induces chemotaxis in target cells, IL-1β, an important mediator of inflammatory response, and TNF-α, which is involved in systemic inflammation and acute phase reaction.

Institutional ethics approval to work with human samples from healthy donors was obtained from the Ethics Committee for Clinical Research (Xunta de Galicia, Spain. 2014/497). All participants included in the study gave their written informed consent.

## Results

### Cell viability

Cells were incubated with different concentrations of Nps (TiO_2_, CeO_2_, Al_2_O_3_ and ZnO) and the proliferation curves obtained are shown in Figure 1.

Cell toxicity was not observed at the concentrations tested using TiO_2_, CeO_2_ or Al_2_O_3_ Nps (Figure 1A) and only a slight reduction in cell proliferation was evident at the highest concentrations, especially for Al_2_O_3_ Nps. However, ZnO Nps were very toxic and they induced cell toxicity with a lethal dose 50 (LD50) of 49.93 μg/ml. According to our results with ZnO Nps, there is a concentration range over which the cell viability is dramatically reduced, with nearly 100% of the cells killed [39]. This finding is consistent with the hypothesis that the release of Zn^2+^ could be the main cause of cell toxicity.

The toxicity of the ZnO Nps was also characterized by performing a colorimetric test in the Jurkat cell line (Supplementary Information). The LD50 in Jurkat cells (Supplementary Figure 1) was close to the value found for the NCI-H460 cell line. In a similar way to this cell line, the other Nps did not have any effect on the cell viability (data not shown).

### ZnO Nps activate MAPK phosphorylation in NCI-H460 cells

The MAPK phosphorylation (p-ERK1/2, p38 and p-SAPK/JNK) was initially measured in order to determine which pathways were activated in NCI-H460 cells by different Nps. When cells were exposed to the Nps for 1 or 3 h, only the ZnO Nps at the highest concentration activated the three different MAPKs (Figure 2). The p-ERK1/2 bands were the most intensely labeled after 1 h, whereas the p-p38 and p-SAPK/JNK levels were stronger after 3 h in the presence of these Nps. By contrast, none of the other metal oxide Nps tested (CeO_2_, TiO_2_ and Al_2_O_3_) activated any MAPK (Figure 2). Longer times in the presence of the CeO_2_ and TiO_2_ Nps (6 h) also failed to produce any positive effect. Likewise, exposure of NCI-H460 cells to a tenfold lower concentration of each Np did not activate any MAPK (Figure 3).

### ZnO & Al_2_O_3_ Nps activate the NFκB pathway

Activation of the NFκB pathway is characterized by degradation of the IκBα inhibitor. However, this degradation was not observed in the NCI-H460 cells incubated with the CeO_2_ and TiO_2_ Nps (100 µg/ml: Figure 2), except for the slight degradation evident in cells maintained for 6 h in the presence of TiO_2_ Nps. By contrast, ZnO (50 µg/ml) and Al_2_O_3_ (100 µg/ml) Nps both altered the amount of IκBα protein in this lung tumor cell line (Figure 2). These Nps increased IκBα protein expression compared with the controls, but this protein almost completely disappeared from cells maintained for 3 h with the ZnO Nps. Conversely, in the presence of Al_2_O_3_ Nps the amount of this protein remained higher than in the control cells. At a concentration ten-times lower, only ZnO Nps induced some degradation of the IκBα protein at both times (Figure 3).

### Effect of Zn^2+^ ions on the activation pathways induced by ZnO Nps

In order to assess whether the effect induced by ZnO Nps on NCI-H460 cells was due to the Np itself or to the action of Zn^2+^ ions alone, the cells were exposed to ZnCl_2_ (1, 10 and 100 µg/ml Zn^2+^ ions) and MAPK and NFκB activation was subsequently assessed in western blots (Figure 4).

Phosphorylation of proteins involved in the activation pathways was observed in the presence of the highest dose of ZnCl_2_ (Figure 4). The phosphorylation of p38 and SAP/JNK proteins was clearly detected after 3 h in the presence of the highest concentration of ZnCl_2_. In the case of p38 some subtle activation was also observed with 10 µg/ml of the zinc salt.

Activation of ERK1/2 was detected in NCI-H460 cells at both time points (1 and 3 h) and this finding is consistent with the results previously found for ZnO Nps (Figure 2). Interestingly, the degradation of IκBα, the inhibitor of the NFκB protein, was also detected in cells exposed to a low salt concentration (1 µg/ml) and the level of degradation increased with both concentration and time of exposure (Figure 4).

### ZnO & Al_2_O_3_ Nps induce secretion of cytokines

In an effort to determine the potential inflammatory and immunogenic properties induced by the metal oxide Nps, a multiplex analysis of 11 cytokines was carried out in human PBMCs. This analysis showed that ZnO and Al_2_O_3_ Nps were the only Nps that induced the release of IL-8 in human PBMCs, and ZnO Nps induced the release of other inflammatory cytokines such as IL-1β, IL-6 and TNF-α (Table 1 and Supplementary Table 1). The CeO_2_ and TiO_2_ Nps induced some cytokine secretion, but only in one or two out of three samples.

## Discussion

The effects induced at the cellular and molecular levels by four common metal oxide Nps (CeO_2_, TiO_2_, Al_2_O_3_ and ZnO) on a lung epithelial cell line were characterized by measuring the cell proliferation and the activation of MAPK and NFκB pathways.

ZnO Nps were the most toxic to the epithelial lung cells (Figure 1) of all the Nps studied and they induced the activation of the three main MAPKs (ERK1/2, p38, SAP/JNK) and the NFκB pathways (Figure 2). Nevertheless, this activation was not detected at the lowest concentration tested, 5 µg/ml, except for the NFκB transcription factor (Figure 3).

The activation of the mainly apoptotic SAP/JNK pathway at the highest concentration is consistent with the cytotoxicity observed for these Nps (Figure 1), which showed an LD50 of approximately 50 µg/ml in the NCI-H460 cell line. A similar cytotoxicity pattern was described for these Nps in different cell lines [33] including Jurkat cells (Supplementary Figure 1).

CeO_2_ Nps were the most innocuous of the four metal oxide Nps studied for the lung cells as they failed to induce activation of any of the signaling pathways at 10 and 100 µg/ml (Figures 2 and 3). TiO_2_ Nps were also unable to induce any of the signaling pathways, except for a slight degradation of the NFκB inhibitor, the IκBα protein, at 6 h (Figure 2).

Al_2_O_3_ Nps increased the expression of the IκBα protein at the highest concentration tested, but not at a concentration that was ten-times lower (Figure 3). This effect could be due to activation of the NFκB pathway as the inhibitor IκBα protein is activated by the nuclear factor in an inhibitory feedback loop [40].

The influence of ion release on the activation routes was also tested for ZnO Nps by using ZnCl_2_. Activation of SAP/JNK was only detected at the highest concentration (100 µg/ml Zn^2+^) and after 3 h of incubation (Figure 4), whereas activation of the protein by the ZnO Nps was already detected at a lower concentration (50 µg/ml) and in a shorter time (1 h) (Figure 2). The p38 and ERK1/2 proteins were activated at both 10 and 100 µg/ml Zn^2+^ although the phosphorylation of p38 was very low at the lower concentration.

Degradation of the IκBα protein was detected at all ion concentrations tested in a similar way to the findings for the ZnO Nps. Hence, Zn^2+^ ions were able to reproduce the cell activation induced by the ZnO Nps. Although there might by other cellular effects due to the Nps themselves, it is probable that the main toxicity mechanism for these Nps is due to the Zn^2+^ ions, as determined previously for other cell lines [34–36]. In addition to their dissolution in the medium, Nps can be further solubilized in the cell lysosomes and this would increase the ion concentration within the cells [36].

ZnO Nps were also able to induce the NFκB pathway at both concentrations tested, thus indicating an inflammatory potential on the lung cells. This activation has also been observed in human bronchial epithelial cells [32]. In fact, ultrafine ZnO particles are capable of reaching the alveoli and they can induce metal-fume fever [41]. Although the use of an epithelial lung cell line is not the optimal model, we selected this type of cell rather than primary cells. The difficulty in obtaining primary human airway epithelial cells and the interindividual variability due to prior activation with other stimuli such as smoking would complicate the characterization.

Activation of the NFκB pathway by the Al_2_O_3_ Nps at the highest concentration tested could also be related to an inflammatory event. For example, Al_2_O_3_ dust has been linked to pulmonary fibrosis in workers exposed to these particulates, and aluminium constituted more than 90% of the metals detected in their lungs [42]. Our results indicate that activation of the different pathways is dose-dependent and such activation was not detected at low concentrations.

In agreement with the activation of the NFκB pathway in the lung cell line, which is mainly linked to inflammation, it was found that ZnO and Al_2_O_3_ Nps were the only Nps that were able to induce IL-8 release in human PBMCs (Table 1 & Supplementary Table 1). In the case of the ZnO Nps, other inflammatory cytokines, such as IL-6, IL-1β and TNF-α, were also produced by these cells. Hence, these Nps have pro-inflammatory properties and are potentially harmful not only for the lung, but also for other tissues and cells. The interaction of the metal oxide Nps with immune cells was studied in a previous work in which human peripheral blood leukocytes from healthy donors were used [43]. The results showed a decrease in the chemotactic response of the leukocytes to SDF-1α in the presence of ZnO Nps, and an increase in the cell proliferation induced by phytohemagglutinin (PHA) in the presence of Al_2_O_3_ and TiO_2_ Nps.

In contrast to ZnO and Al_2_O_3_, CeO_2_ and TiO_2_ Nps did not show any harmful effects toward the lung epithelial cells and only TiO_2_, at the highest concentration and longest incubation time tested, seemed to induce some degradation of IκBα, the NFκB inhibitor. Nevertheless, TiO_2_ and CeO_2_ Nps produced some cytokines for only one or two in three samples from healthy volunteers. Hence, although these Nps are apparently safe for epithelial lung cells, they potentially could have inflammatory or allergenic properties for some individuals.

## Conclusion

A comparative study of human lung epithelial cell activation by four different metal oxides (CeO_2_, TiO_2_, Al_2_O_3_ and ZnO), using the NCI-H460 cell line, showed that only ZnO Nps were able to induce activation of the ERK, p38 and SAP/JNK kinases, as characterized by detection of the phosphorylated protein using western blot. Activation of the mainly apoptotic SAP/JNK kinases is consistent with the cytotoxic potential of these Nps. Moreover, cells in the presence of ZnCl_2_ – as a source of Zn^2+^ ions – gave a similar activation pattern as the cells incubated with ZnO NPs, which suggests that the ions released by the ZnO Nps are mainly responsible for the effects observed on the cells. Both ZnO and Al_2_O_3_ Nps activated mainly inflammatory NFκB pathway (studying the degradation of the IκBα inhibitor). This pro-inflammatory character was further confirmed by the cytokine profile obtained in human PBMCs incubated with these Nps. Finally, CeO_2_ and TiO_2_ Nps showed a much safer profile, with only minor effects observed on the aforementioned cells.

## Future perspective

The results of the study described here demonstrate that Nps can activate different cell signaling pathways. Analysis of the possible activation of these routes is also important to understand the effect that the Nps have on the cells besides toxicity, ROS generation or genotoxicity.

The characterization of the cell activation at the molecular level will allow a better understanding of the mechanism by which Nps can exert harmful effects. Each type of Np would require an independent study because any subtle variation could induce a marked difference in how the Nps interact with the cells, but over a long period these studies could provide relevant information on the potential effects *in vivo*. In fact, one of the main problems currently faced in the field of nanotoxicology is the lack of standardized *in vitro* methods to study the increased variety of Nps that are already being produced, and the poor correlation between the effects observed *in vitro* and *in vivo*. The characterization of the activation at the molecular level could contribute to the identification of parameters related with physiological or pathological processes. The activation of signaling proteins could be one of these key parameters that should be characterized in different cell types to predict the potential harmful or beneficial effects of any type of Np. The development of new and faster techniques to measure the activation of a large number of signaling proteins in a single experiment would allow these types of assays to be included in routine nanotoxicology studies.

**Table 1.** **Cytokine release after incubation of human peripheral blood mononuclear cells with the different metal oxide Nps at two different concentrations: 20 and 200 µg/ml.**

		**μg/ml**	**ZnO**	**TiO_2_**	**CeO_2_**	**Al_2_O_3_**
Th1 profile	IL 2	20	-	-	+1/3	-
		200	-	+1/3	-	+1/3
	IFN-γ	20	-	+1/3	+1/3	-
		200	-	-	-	-
	IL-12p70	20	-	-	-	-
		200	-	-	-	-
	TNF-β	20	-	-	-	-
		200	-	-	-	-
Th2 profile	IL4	20	-	-	-	-
		200	-	-	-	-
	IL5	20	-	-	-	-
		200	-	-	-	-
	IL6	20	+	-	-	-
		200	+	-	-	+ 1/3
	IL10	20	-	+1/3	-	-
		200	-	+1/3	+ 1/3	+1/3
Other pro-inflammatory cytokines	IL8	20	++	-	-	+
		200	++	-	-	+
	IL1β	20	+	+1/3	+2/3	-
		200	+	+1/3	+ 1/3	+2/3
	TNF-α	20	+	-	+ 1/3	-
		200	+	+1/3	+1/3	+ 1/3

-: Cytokine concentration similar to the negative control.

+: Cytokine concentration between the negative control and the positive control and at least ten-times or higher than the detection limit.

++: Cytokine concentration at or above the positive control.

+ 1/3: One out of three donors is positive.

**Executive summary**

Metal oxide Nps are widely used in several industrial processes and products such as catalysts, cosmetics, coatings and also in the biomedical field.
Nps can reach the lung by intentional administration or by accidental exposure and their toxic effects have not been fully characterized.
At a cellular level, Nps can stimulate the activation of different signaling pathways related with proliferation, inflammation and apoptosis.
The potential activation of human lung epithelial cells by four different metal oxide Nps was studied in the NCI-H460 cell line, and the activation of the three main MAPKs and NFκB was characterized by western blot.
Cell viability was also studied using a method based on the changes in cell impedance. Only ZnO Nps reduced the viability of the cells and this finding is consistent with other studies that have shown the cytotoxicity of ZnO Nps in different cell types.
ZnO Nps were able to activate the three MAPKs studied (ERK, p38 and SAP/JNK), and the release of Zn^2+^ ions seems to be the main cause of this effect. These results are consistent with their potential cytotoxicity.
ZnO and Al_2_O_3_ Nps showed inflammatory properties due to activation of the NFκB pathway in lung epithelial cells and the release of pro-inflammatory cytokines in human PBMCs.
TiO_2_ and CeO_2_ exhibited safer profiles in the lung cells, with only minor effects observed after treatment with this type of Nps. Nevertheless, the capacity of TiO_2_ Nps to increase the proliferation of activated T lymphocytes was shown in a previous work.
