# Supplementary material for: Analysis of the activation routes induced by different metal oxide nanoparticles on human lung epithelial cells
Source: Future Sci OA. 2016 Apr 15;2(2):FSO118. doi: 10.4155/fso.16.2 (PMC5137956; doi:10.4155/fso.16.2)
Supplement: Supplementary file 1 [file fso-02-118-s1.docx]

| **pg/ml** | Treatment | **IL-12p70** | **IFN-gamma** | | **IL-2** | **IL-10** | **IL-8** | **IL-6** | **IL-4** | **IL-5** | **IL-1beta** | **TNF-alpha** | **TNF-beta** |
| --- | --- | --- | --- | --- | --- | --- | --- | --- | --- | --- | --- | --- | --- |
| Donor 1 | **NC** | 0 | | 0 | 0 | 0 | 1038.02 | 0 | 0 | 0 | 0 | 0 | 0 |
|  | **PC** | 99.27 | | 816 | 577.385 | 549.82 | 3687.89 | 5823.67 | 0 | 0 | 1433.58 | 5627.08 | 3.81 |
|  | **ZnO 20** | 0 | | 0 | 0 | 0 | 4223.71 | 1398.12 | 0 | 0 | 384.995 | 725.28 | 0 |
|  | **ZnO 200** | 0 | | 0 | 0 | 0 | 4440.985 | 3290.965 | 0 | 0 | 681.575 | 1708.145 | 0 |
|  | **TiO_2_ 20** | 0 | | 0 | 0 | 0 | 1291.86 | 0 | 0 | 0 | 0 | 0 | 0 |
|  | **TiO_2_ 200** | 2.2 | | 11.415 | 140.85 | 39.645 | 1085.84 | 0 | 114.56 | 46.6 | 223.31 | 56.76 | 36.355 |
|  | **CeO_2_ 20** | 0 | | 0 | 0 | 13.17 | 1065.435 | 0 | 58.93 | 11.665 | 79.635 | 27.205 | 12.895 |
|  | **CeO_2_ 200** | 0 | | 0 | 0 | 0 | 1185.97 | 0 | 0 | 0 | 0 | 0 | 0 |
|  | **Al_2_O_3_ 20** | 0 | | 0 | 0 | 0 | 1824.375 | 0 | 0 | 0 | 0 | 0 | 0 |
|  | **Al_2_O_3_ 200** | 2.67 | | 33.275 | 129.425 | 40.285 | 4760.695 | 181.84 | 125.8 | 48.775 | 483.46 | 341.785 | 40.875 |
| Donor 2 | **NC** | 0 | | 0 | 0 | 0 | 487.245 | 0 | 0 | 0 | 0 | 0 | 0 |
|  | **PC** | 105.3 | | 700.14 | 0 | 63.05 | 4121.48 | 2880.555 | 0 | 0 | 1042.175 | 3896.705 | 0 |
|  | **ZnO 20** | 0 | | 0 | 0 | 7.995 | 3478.3 | 974.705 | 15.835 | 0 | 490.1 | 634.135 | 0 |
|  | **ZnO 200** | 0 | | 0 | 0 | 0 | 2851.47 | 1399.365 | 0 | 0 | 335.62 | 978.035 | 0 |
|  | **TiO_2_ 20** | 5.17 | | 56.115 | 135.205 | 33.355 | 563.08 | 0 | 125.8 | 57.635 | 276.415 | 70.45 | 54.855 |
|  | **TiO_2_ 200** | 0 | | 0 | 0 | 0 | 441.525 | 0 | 0 | 0 | 0 | 2.2 | 0 |
|  | **CeO_2_ 20** | 0 | | 0 | 0 | 0 | 494.445 | 0 | 0 | 0 | 0 | 2.2 | 0 |
|  | **CeO_2_ 200** | 0 | | 14.725 | 98.835 | 23.465 | 570.72 | 0 | 120.2 | 26.265 | 118.435 | 54.425 | 24.32 |
|  | **Al_2_O_3_ 20** | 0 | | 0 | 0 | 0 | 684.82 | 0 | 0 | 0 | 0 | 0 | 0 |
|  | **Al_2_O_3_ 200** | 0 | | 0 | 0 | 0.115 | 1775.485 | 8.13 | 0 | 0 | 21.195 | 17.67 | 0 |
| Donor 3 | **NC** | 0 | | 0 | 0 | 4.505 | 969.475 | 0 | 48.9 | 11.665 | 44.985 | 20.225 | 6.935 |
|  | **PC** | 16.13 | | 819.925 | 169.615 | 524.095 | 2841.315 | 5144.015 | 0 | 0 | 1605.51 | 4699.025 | 0 |
|  | **ZnO 20** | 0 | | 0 | 33.985 | 7.415 | 3585.715 | 450.565 | 65.795 | 16.05 | 149.345 | 201.415 | 12.895 |
|  | **ZnO 200** | 0 | | 0 | 0 | 0 | 3783.24 | 1242.395 | 0 | 0 | 459.42 | 678.85 | 0 |
|  | **TiO_2_ 20** | 0 | | 0 | 0 | 7.82 | 1029.855 | 0 | 42.985 | 0 | 0 | 22.105 | 0 |
|  | **TiO_2_ 200** | 0 | | 0 | 0 | 0 | 790.26 | 0 | 0 | 0 | 0 | 0 | 0 |
|  | **CeO_2_ 20** | 0 | | 19.43 | 95.62 | 16.63 | 746.41 | 0 | 87.275 | 35.855 | 143.155 | 50.71 | 36.355 |
|  | **CeO_2_ 200** | 0 | | 0 | 0 | 0 | 794.365 | 0 | 0 | 0 | 0 | 0 | 0 |
|  | **Al_2_O_3_ 20** | 0 | | 0 | 0 | 0 | 1094.76 | 0 | 0 | 0 | 0 | 0 | 0 |
|  | **Al_2_O_3_ 200** | 0 | | 0 | 0 | 0 | 2268.12 | 0 | 0 | 0 | 0 | 1.415 | 0 |
| Detection limit | | 1.5 | | 1.6 | 16.4 | 1.9 | 0.5 | 1.2 | 20.8 | 1.6 | 4.2 | 3.2 | 2.4 |
