# Supplementary material for: Analysis of the activation routes induced by different metal oxide nanoparticles on human lung epithelial cells
Source: Future Sci OA. 2016 Apr 15;2(2):FSO118. doi: 10.4155/fso.16.2 (PMC5137956; doi:10.4155/fso.16.2)
Supplement: Supplementary file 2 [file fso-02-118-s2.docx]

**Figure S1. Effect of ZnO Nps on the viability of Jurkat cells.**

The cell viability was characterized by measuring the cell metabolic activity of Jurkat cells with a colorimetric method based on a tetrazolium compound, the namely MTS. The LD50 was 55 ± 5 µg/mL.

**Table S1. Cytokine concentration in the supernatant of human PBMCs after incubation with the metal oxide nanoparticles at two different concentrations.**

Peripheral blood mononuclear cells were obtained from three healthy donors, and incubated at a cell density of 1 × 10^6^ / ml for 24 hours with the Nps (at 20 or 200 µg/ml). Cells incubated with culture medium or in the presence of LPS (1 µg/ml) plus PHA (10 µg/ml), were used as negative (NC) and positive controls (PC), respectively.
